# Metabolic rewiring in the promotion of cancer metastasis: mechanisms and therapeutic implications

**DOI:** 10.1038/s41388-020-01432-7

**Published:** 2020-08-24

**Authors:** Qinyao Wei, Yun Qian, Jun Yu, Chi Chun Wong

**Affiliations:** 1 Institute of Digestive Disease and Department of Medicine and Therapeutics, State Key Laboratory of Digestive Disease, Li Ka Shing Institute of Health Sciences, CUHK Shenzhen Research Institute, Chinese University of Hong Kong, Hong Kong, China; 2 Department of Gastroenterology and Hepatology, Shenzhen University General Hospital, Shenzhen, China

**Keywords:** Cancer metabolism, Metastasis

## Abstract

Tumor metastasis is the major cause of mortality from cancer. Metabolic rewiring and the metastatic cascade are highly intertwined, co-operating to promote multiple steps of cancer metastasis. Metabolites generated by cancer cells influence the metastatic cascade, encompassing epithelial-mesenchymal transition (EMT), survival of cancer cells in circulation, and metastatic colonization at distant sites. A variety of molecular mechanisms underlie the prometastatic effect of tumor-derived metabolites, such as epigenetic deregulation, induction of matrix metalloproteinases (MMPs), promotion of cancer stemness, and alleviation of oxidative stress. Conversely, metastatic signaling regulates expression and activity of rate-limiting metabolic enzymes to generate prometastatic metabolites thereby reinforcing the metastasis cascade. Understanding the complex interplay between metabolism and metastasis could unravel novel molecular targets, whose intervention could lead to improvements in the treatment of cancer. In this review, we summarized the recent discoveries involving metabolism and tumor metastasis, and emphasized the promising molecular targets, with an update on the development of small molecule or biologic inhibitors against these aberrant situations in cancer.

## Introduction

It has been appreciated since the early days of oncology research that the metabolic profiles of tumor cells differ from normal cells. Thompson et al. organized known cancer-associated metabolic phenotypes into six hallmarks: (1) dysregulated uptake of glucose and amino acids, (2) use of opportunistic modes of nutrient acquisition, (3) use of glycolysis/TCA cycle intermediates for biosynthesis and NADPH production, (4) increased demand for nitrogen, (5) alterations in metabolite-driven gene regulation, and (6) metabolic interactions with the microenvironment [1]. Cancer cells have high metabolic demands and rewire cellular metabolism to support malignant properties.

Cancer metastasis is the primary cause of cancer-associated mortality. However, our current understanding of the biology of metastases and development of druggable targets against tumor metastasis lags behind that for primary cancers. Metastasis is a multistep process consisting of a series of sequential events: (1) local invasion into the tumor-associated stroma, (2) intravasation into hematopoietic, lymphatic systems or peritoneum, (3) survival in circulation, (4) extravasation into pre-metastatic niches at distant organs, and (5) colonization to form metastases [2, 3]. Metastatic sites often present different metabolic challenges for the cancer cells with respect to nutrient and oxygen availability, as well as oxidative stress [4].

**Fig. 1:**
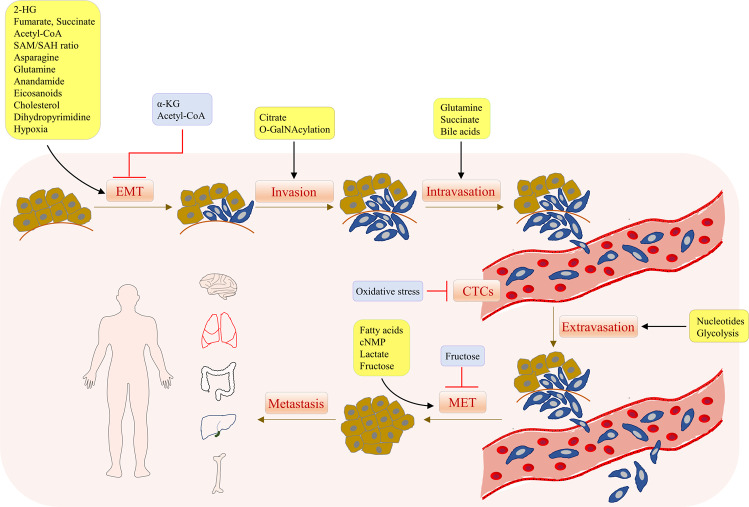
Metabolites modulate the tumor metastasis cascade. Overall summary of the role of metabolites in different stages of the tumor metastasis cascade, including epithelial-mesenchymal transition (EMT), invasion, intravasation, survival in circulation, extravasation and outgrowth into detectable metastasis.

Emerging evidence indicates a complex interplay between tumor metastasis and metabolic rewiring in cancer (Fig. 1). Metabolic rewiring could drive metastasis by (1) generating oncometabolites to hijack metastatic signaling cascades via regulating gene expression, (2) generating metabolites/cofactors that act as agonists/antagonists for functional proteins involved in metastasis, and (3) modulating metabolic demands of cancer cells, thus allowing adaptation to the various stages of metastatic cascade. On the other hand, metastatic-associated signaling can influence cellular metabolism by directly affecting the expression and activity of metabolic enzymes. In this review, we provide a summary of molecular mechanisms linking cancer metastasis and cell metabolism, their underlying roles in metastasis and highlight the potential metabolic targets that could be harnessed to suppress cancer metastasis.

## Effects of metabolic rewiring on metastatic signaling cascades in cancer

### Metabolites regulating epithelial-mesenchymal transition to facilitate tumor invasion

Epithelial-Mesenchymal transition (EMT) is a critical initiating step in the metastatic cascades. Epithelial cells that underwent EMT lose their polarity and gain the ability to infiltrate neighboring tumor-associated stroma. EMT is characterized by loss of epithelial marker E-cadherin, together with increased mesenchymal markers vimentin and N-cadherin [5]. EMT is primarily driven by the transcription factors SNAIL1/2, zinc-finger E-box-binding (ZEB1/2), and basic helix-loop-helix transcription factors (TWIST1/2) that repress epithelial marker genes whilst inducing expression of mesenchymal markers. These transcription factors are overexpressed in response to extracellular mitogenic signals. Tumor microenvironment, such as inflammation and hypoxia [5, 6], are also triggers for EMT. Below we outlined metabolites that could modulate EMT and drives the initial steps of metastatic progression.

#### 2-hydroxyglutarate (2-HG)

2-HG is an oncometabolite identified to accumulate in tumors harboring mutations in isocitrate dehydrogenase 1 or 2 (IDH1/2). IDH1/2 are TCA cycle enzymes that catalyzes a two-step reaction for oxidative decarboxylation of isocitrate to α-ketoglutarate (α-KG). IDH1 (R132) or IDH2 (R140/R172) mutations abolish their original catalytic activity but are accompanied with gain-of-function in conversion of α-KG to 2-HG, leading to very high 2-HG levels (5–35 mM) [7]. Some IDH1/2-wildtype tumors evolved alternative mechanisms for 2-HG production. In breast cancer, glutaminolysis drives L-2-HG biosynthesis via wildtype IDH2. L-2-HG can also be derived from α-KG via lactate dehydrogenase 1 or malate dehydrogenase at acidic pH [8]. Besides, deficiency of 2-hydroxyglutarate dehydrogenases elevates 2-HG by preventing its conversion back to α-KG [8].

2-HG is structurally similar to α-KG and behaves as a competitive antagonist to inhibit α-KG-dependent dioxygenases (>60) involved in diverse biological functions [9, 10]. The roles of 2-HG in epigenetic deregulation have received intensive attention. 2-HG inhibits the activity of DNA demethylases (Ten-eleven Translocation, TET) and histone demethylases (Jumonji C, e.g., KDMs), leading to aberrant DNA and histone methylation. These α-KG-dependent dioxygenases have K_m_ for α-KG at physiological concentrations, making their activities susceptible to fluctuations in α-KG or 2-HG [11]. Recent work has shown that 2-HG-triggered DNA and histone methylation are involved in tumor metastasis.

2-HG is an inducer ZEB1-mediated EMT [12]. Comparison of isogenic cell lines expressing mutant or wildtype IDH1/2 revealed that cancer cells expressing mutant IDH1/2 exhibited reversible EMT phenotype with a fibroblast-like morphology, along with reduced E-cadherin but increased levels of fibronectin, vimentin and N-cadherin. Exogenous 2-HG recapitulated induction of EMT in IDH1/2 wildtype cells, implying a direct role for this metabolite in EMT. ZEB1 knockdown or miR200 re-expression reverted IDH1/2-mutant cells to an epithelial phenotype, suggesting that the effect of 2-HG on EMT is dependent on ZEB1/miR-200 pathway. Colvin et al. [8] showed that D-2HG, but not L-2HG, increased the trimethylation of H3K4, an active transcription mark, at ZEB1 promoter, leading to ZEB1 expression and EMT. Accordingly, higher D-2HG in colorectal tumors was associated with distant organ metastasis. 2-HG thus promotes a prometastatic phenotype via epigenetic activation of ZEB expression.

#### Succinate and fumarate

Succinate and fumarate are also competitive antagonists for α-KG-dependent dioxygenases. Inactivating mutations of fumarate hydratase (FH) and succinate dehydrogenase (SDH) drive the accumulation of fumarate and succinate, respectively. SDH catalyzes the oxidation of succinate to fumarate. Mutations in SDH complex are found in gastrointestinal stromal tumors, renal cell carcinoma, phaeochromocytomas syndrome (PCC), or paraganglioma (PGL), leading to succinate accumulation [9]. FH mediates reversible conversion between fumarate and malate. FH mutations can be found in renal cell carcinoma, where its loss-of-function leads to elevated levels of fumarate [9].

Recent evidence showed that succinate and fumarate accumulation contribute to SNAIL-, TWIST- and ZEB-mediated EMT via the inhibition of DNA and/or histone demethylases. In PCC, PGL and ovarian cancers, SDHB mutation or knockdown led to histone methylation and increased expression of EMT markers LOXL2, TWIST1/2, TCF3, and metalloproteases (MMPs) [13–15]. In CRC cells, SDHB knockdown also activated TGF-β signaling and SNAIL-dependent EMT [16]. Sciacovelli et al. [17] revealed that FH-deficient cells displayed features of EMT. Mechanistically, fumarate suppresses DNA demethylases, resulting in CpG methylation of an antimetastatic microRNA-200ba429 cluster. Transcriptional silencing of miR-200 cluster induced expression of vimentin, SNAIL2, ZEB1, and ZEB2. Apart from epigenetic alterations, increased fumarate upon FH loss in hereditary leiomyomatosis and renal cell cancer (HLRCC) modulated NFE2-related factor 2, which promoted EMT via activation of NOTCH1 [18–20]. Indeed, FH-deficient HLRCC is associated with tumor metastasis. Fumarate and succinate are thus pro-metastastic metabolites that drives EMT through epigenetic dysregulation and activation of EMT-associated signaling pathways.

#### α-Ketoglutarate

α-KG functions as a metastasis suppressor through counteracting effects of 2-HG, succinate, or fumarate. α-KG dehydrogenase complex is the key modulator of cellular α-KG by catalyzing conversion of α-KG to succinyl-CoA. Up-regulation of α-KG dehydrogenase in breast cancer confers oncogenic properties as it diminished α-KG levels. Conversely, inhibition of this enzyme mediate α-KG accumulation to suppress tumor progression and metastasis in p53^−/−^ pancreatic ductal adenocarcinoma and breast cancer [21, 22]. Mechanistically, α-KG promotes TETs-mediated promoter demethylation and re-expression of antimetastatic miR-200 cluster, thereby down-regulating ZEB1/CtBP1-MMP3 axis and EMT phenotype [22]. Collectively, these studies highlight the α-KG-to-succinate/fumarate/2-HG ratios as pivotal regulators of EMT phenotype and metastasis in multiple cancer types.

#### Acetyl-CoA

Acetyl-CoA is an important intermediate at the crossroads of glycolysis, glutaminolysis, and fatty acid metabolism. Cytosolic and nuclear acetyl-CoA levels are maintained by four metabolic pathways: (1) direct synthesis from acetate and CoA via acetyl-CoA synthetase short-chain family (ACSS); (2) conversion from citrate to acetyl-CoA by ATP citrate lyase (ACLY); (3) its catabolism to acetate and CoA by acyl-CoA thioesterase (ACOT); and (4) irreversible carboxylation from Acetyl-CoA to malonyl-CoA by Acetyl-CoA carboxylase (ACC) [9]. Deregulation of acetyl-CoA has been reported in multiple cancers [23–33], and it contribute to malignant phenotypes.

Acetyl-CoA is involved in epigenetic regulation by functioning as a cofactor for histone acetyltransferase (HATs). Intracellular acetyl-CoA levels fall within K_m_ range of HATs. Increased Acetyl-CoA triggers metastasis by inducing histone acetylation, leading to an open chromatin permissive for gene expression. Acetyl-CoA was shown to be involved in hepatocellular carcinoma (HCC) metastasis [24]. ACOT12, which catabolizes acetyl-CoA, was silenced in HCC and associated with metastasis and poor prognosis [24]. In HCC cells, ACOT12 knockdown induced acetyl-CoA levels, which promoted histone acetylation via KAT2A to induce TWIST2 expression. TWIST2 in turn up-regulated N-cadherin and reduced E-cadherin, thereby contributing to EMT and metastasis in animal models. Acetate addition to boost acetyl-CoA also promoted TWIST2-mediated EMT, underscoring the role of this metabolite in HCC metastasis [24]. In breast cancer, TGF-β signaling was shown to induce ACC1 phosphorylation and inactivation, thus, preventing acetyl-CoA flux toward fatty acid biosynthesis and resulting in elevated acetyl-CoA [26, 34]. Acetyl-CoA directly promoted acetylation and nuclear translocation of Smad2 transcription factor to mediate SNAIL1/2, EMT, and tumor metastasis. In line with the role of acetyl-CoA in EMT induction, silencing of ACLY to abolish acetyl-CoA biosynthesis was sufficient to reverse EMT markers induced by ACC1 silencing, accompanied by blunted invasive capacity [26]. Contrary to the above results, ACSS2 overexpression in HCC was found to mediate acetylation of HIF-2α to inhibit EMT under hypoxia [35]. Therefore, the effect of acetyl-CoA is dependent on its target proteins and the tumor microenvironment.

#### SAM/SAH ratio

DNA (DNMTs) and histone methyltransferases (HMTs) utilize a common methyl donor: S-adenosylmethionine (SAM), a product of one-carbon cycle. Donation of methyl group from SAM releases S-adenosyl-homocysteine (SAH), an inhibitor of DNMTs/HMTs. SAM/SAH ratio dictates methyltransferase activities [9].

SAM was shown to suppress tumor cell invasion in vitro and tumor metastasis in vivo by inducing promoter methylation and silencing of prometastatic genes such as urokinase-type plasminogen activator (uPA) [36]. This inhibited the activation of its receptor uPAR and downstream ERK, PI3K/Akt, Src, and Rac1 [36]. SAM/SAH ratio also influences histone methylation. Nicotinamide N-methyltransferase (NNMT) requires SAM as a methyl donor for converting nicotinamide to 1-methylnicotinamide. NNMT overexpression resulted in SAM depletion and substantially inhibited histone methylation at H3K4, H3K9, H3K27, and H4K20 [9]. Decreased histone methylation then regulated signaling pathways associated with acquisition of a more aggressive phenotype. Consistently, NNMT knockdown decreased expression of EMT-related genes MMP9, SNAI2, vimentin, WNT5B, ZEB1/2; and suppressed cell invasion and migration in squamous cell carcinoma [37]. Aberrant methylation of DNA/histone driven by SAM thus has contrasting effects on EMT to that induced by demethylation blockade via 2-HG/succinate/fumarate.

#### Asparagine and glutamine

Asparagine and glutamine are nonessential amino acids; however, cancer cells demonstrate acquired dependence on these two amino acids. Asparagine synthetase (ASNS) mediates the *de novo* synthesis of asparagine from aspartate and it is up-regulated in multiple cancers [38]. Utilizing a focused shRNA library, Knott et al. [39] identified ASNS as the top essential gene for 4T1 breast cancer cell migration in vitro and lung metastasis in vivo. Silencing of ASNS reduced intracellular asparagine and suppressed cell invasion, an effect rescued by asparagine. Asparagine was shown to promote EMT via up-regulation of TWIST. Treatment with L-asparaginase or dietary asparagine restriction suppressed breast cancer metastasis in vivo, whilst excess dietary asparagine or ASNS ectopic expression exacerbated tumor metastasis. The effect of asparagine is specific to metastasis, as growth of the primary tumor was not affected.

Asparagine also facilitates the expression of glutamine synthetase (GLUL), which sustains cell proliferation and protein synthesis by de novo glutamine biosynthesis [40]. While glutamine is readily available in the circulation, its levels in metastatic niche is low. By promoting GLUL, asparagine mediated tumor cell survival in the distant organs and promoted outgrowth to form metastasis [40, 41]. Up- regulation of GLUL could also directly induce EMT in HCC [42]. Hence, asparagine and glutamine might function co-operatively to fuel tumor metastasis.

#### Anandamide

Anandamide (AEA) is an unsaturated fatty acid derivative derived from arachidonic acid (AA). AEA turnover is regulated by fatty acid amide hydrolase (FAAH) that degrades AEA to ethanolamine and AA. FAAH is up-regulated in CRC, prostate, and lung cancers, and it drives tumorigenic phenotypes [43]. Inhibition of FAAH or AEA addition exerted an inhibitory effect on Wnt/β-catenin mediated EMT in breast cancer, suggesting AEA as an antimetastatic metabolite [44].

#### Eicosanoids

Eicosanoids such as prostaglandins, thromboxanes, leukotrienes, lipoxins, HETEs and EETs, are signaling molecules derived from AA via the action of cyclo-oxygenases (COX), lipoxygenases and cytochrome P450 epoxygenases. As ligands of peroxisome proliferators-activated receptors (PPAR), AEA, and eicosanoids both activate PPARα, PPARβ/δ, and PPARγ, which bind to peroxisome proliferator hormone response elements (PPREs) of SNAILs, ZEBs or TWISTs to regulate their expression and control tumor metastasis [45–52].

Prostaglandin E_2_ (PGE_2_) is a key proinflammatory PG. PGE_2_ upregulated TAMs and MDSCs contributed to immunosuppression and EMT-mediated lung metastasis to mouse lungs [53]. Apart from modulating EMT, PGE_2_ derived from COX-2 induced expression of MIR675–5p, which inhibited p53 and promoted CRC metastasis [54]. PGE_2_ is involved in metastatic lymphangiogenesis in breast cancer [55]. PGE_2_ also induced CSC markers expression and promoted EP4/NF-κB-mediated liver metastasis [56]. PGE_2_ thus have diverse effects on different stages of metastasis. Consistent with prometastatic effect of PGE_2_, combination of a selective COX-2 inhibitor, celecoxib, and VEGF inhibitor Axitinib, caused a striking decrease in the metastasis of HCT116 cells to the liver in mice models [57].

#### Cholesterol

Cholesterol, together with lipids, forms lipid rafts on the cell membrane to regulate the activities of cell surface receptors. Reduced membrane fluidity due to altered cholesterol flux reduced cell motility, stem cell-like properties, and EMT, thus, suppressing tumor cell metastasis in vivo [58]. Concordantly, cholesterol treatment in vitro induced mesenchymal-like morphological features of metastatic prostate cancer cells in vitro, and cholesterol-fed mice formed markedly more liver metastatic nodules than mice fed normal chow diet [59]. Mechanistically, cholesterol induced lipid-rafts stabilize adipocyte plasma membrane-associated protein, which together with epidermal 35 growth factor receptor substrate 15-related protein (EPS15R), reduced endocytosis-mediated EGFR degradation. EGFR in turn activated ERK1/2 to trigger EMT. Targeting of HMG-CoA reductase (HMGCR), a rate-limiting enzyme for cholesterol biosynthesis, greatly down-regulated spontaneous lung metastasis by suppressing expression of MMPs [60].

#### Dihydropyrimidine

Dihydropyrimidine dehydrogenase (DPYD) is the rate limiting enzyme for pyrimidine degradation by catalyzing the breakdown of uracil/thymine to dihydrouracil [DHU] and dihydrothymine [DHT]. A comprehensive analysis of 1704 metabolic genes in 978 human cancer cells, followed by functional analysis using an RNAi library identified that DPYD is associated with a mesenchymal phenotype [61]. DPYD depletion abrogated EMT in HMLE cells expressing Twist, as exemplified by suppression of mesenchymal markers Vimentin, N-cadherin, ZEB1/2, and SNAIL1/2. DPYD knockdown abrogated EMT-induced invasion of lung tissues by HMLE-Twist cells, suggesting that DPYD is essential for EMT-induced metastasis. Notably, DPYD positively regulated intracellular dihydropyrimidine-to-pyrimidine ratio that correlates with mesenchymal character. Dihydropyrimidines supplementation restored EMT in DPYD depleted cells, confirming their direct involvement in EMT [61]. DPYD was overexpressed in HCC cells with high metastatic potential, and gain-/loss-of-function studies confirmed the role of DPYD in HCC metastasis [62]. Consistently, expression of DPYD is associated with poor survival of HCC patients. Nevertheless, mechanisms whereby dihydropyrimidine induce EMT remains unknown. Further investigation is required to identify the direct molecular target of these metabolites.

#### Hypoxia

In solid tumors, hypoxia, or low oxygen tension, is a hallmark feature of the tumor microenvironment arising from an imbalance between increased consumption of oxygen by cancer cells combined with inadequate oxygen supply. In response to hypoxia, hypoxia-inducible factors (HIFs) and their downstream gene networks are triggered to allow adaptation to low oxygen conditions [63, 64]. HIF-1α is a driving factor for tumor angiogenesis and mediates EMT-associated metastasis [63]. HIF-1α induced EMT through increased expression of vimentin, fibronectin, N-cadherin, snail, twist, TIMP-1 and -2, and pERK in mouse fibroblasts [65]. HIF-1 transactivates EMT inducing factors, TWIST and ZEB1, by binding directly to hypoxia-response element (HRE) in the promoters of TWIST and ZEB1 [66, 67]. Repression of TWIST under hypoxia or in HIF1α-overexpressing cells prevented EMT and abolished metastatic phenotypes [66, 68]. ZEB1 inhibition abrogated HIF-1α-induced EMT and invasion in colorectal cancer cells [67], inferring a critical role of this transcription factor in mediating its prometastatic effect. Moreover, HIF signaling indirectly promotes EMT through induction of Notch, TGF-β, integrin-linked kinase, Wnt, Hedgehog, and AXL receptor tyrosine kinase signaling [63]. Accordingly, hypoxia and HIF-1α are major promoting factors in EMT-mediated metastasis.

### Metabolites that modulate MMPs and invasive front infiltration

Matrix metalloproteinase (MMP) is one of major players to facilitate local invasion and invasive front infiltration [3]. The endothelial basement membrane (BM) presents a mechanical barrier that prohibits cancer cells infiltration into neighboring stroma and extravasation after cell engraftment at distant organs [3]. MMPs, proteinases that degrade ECM proteins, are crucially involved in overcoming BM barrier [69]. Cancer cells up-regulate MMPs to promote uncontrolled proteolysis and degradation of BM, thereby promoting infiltration [3]. Citrate and O-Glycosylation are two metabolites that modulate MMPs to promote metastasis [3, 70, 71], as described below.

#### Citrate

Up-regulation of phosphofructokinase (PFKP) and citrate synthase (CS) were found to enhance the activity and expression of MMP2 and MMP9 to promote breast cancer metastasis [71]. PFKP induced glycolysis, which in concert with CS activity to generate excess intracellular citrate. Citrate accumulation via PFKP, CS, or exogenous citrate triggers AKT/ERK signaling to up-regulate MMP2 and MMP9 expression, leading to enhanced cell invasion and metastasis of triple-negative breast cancer [71].

#### O-GalNAcylation

O-GalNAcylation is a post-translational modification critical for MMPs activity. O-N-acetylgalactosamine transferases (GALNTs), which is frequently overexpressed in cancers [70, 72], catalyzes protein O-GalNAcylation involving the attachment of O-linked GalNAc to serine or threonine residues of secreted proteins or cell surface mucins to form N-acetylgalactosamine (Tn carbohydrate antigen) [73, 74]. The core 1 synthase, glycoprotein-N-acetylgalactosamine 3-beta-galactosyltransferase 1 (C1GALT1) catalyzes the second step of O-glycosylation by addition of galactose to Tn that forms core 1 carbohydrate structure [73]. Contrary to GALNTs, C1GALT1 is inactivated in cancer [72–75]. Increased GALNTs or the loss of C1GALT1 cause enhanced O-GalNAcylation and Tn in tumors [70], which in turn modulates MMPs [76]. Overexpression of GALNT14 resulted in up-regulation of MMP-2 in MCF-7 cells, while silencing of GALNT14 suppressed MMP-2 [72]. O-GalNAcylation also modifies MMP-14 activity. In liver tumors, expression of an ER-targeted GALNT1 increased Tn and enabled efficient matrix degradation and tissue invasion of cancer cells via O-GalNAcylation of MMP14 [70]. Both elevated GALNTs or dysregulated C1GALT1 could lead to hyper-O-GalNAcylation of MMPs and are common features of metastatic cancers.

### Pro-angiogenesis metabolites promoting intravasation into hematopoietic systems

Intravasation is governed by invasive capacities of cancer cells and tumor-associated angiogenesis, which are typically leaky with high permeability. Hypoxic and nutrient deficient microenvironment is a major trigger for tumor-driven angiogenesis [77]. Succinate and glutamine are metabolites with known associations with angiogenesis.

#### Glutamine

M2-like macrophages (TAMs) are commonly associated with tumors and they possess pro-angiogenic, immunosuppressive and metastatic function. Palmieri et al. [78] demonstrated that glutamine metabolism in macrophages plays a key role in macrophage polarization and promotion of tumor metastasis. Inhibition of glutamine synthetase (GS) reversed M2 phenotype (TAMs) and promoted polarization to the M1 phenotype. GS blockade reduced intracellular glutamine, and glutamine uptake was channeled toward succinate biosynthesis through gamma-aminobutyric acid shunt. Succinate stabilized HIF-1α to enhance glycolysis flux and promote reversion to M1 phenotype. Macrophage-specific deletion of GS in macrophages promoted cytotoxic T-cell infiltration, reduced angiogenesis, and suppressed tumor metastasis, indicating that targeting GS in macrophages might be useful for metastasis treatment.

#### Succinate

In contrast to its antimetastatic role in macrophages, succinate promotes angiogenesis via two mechanisms. First is by stabilization of HIF in cancer cells through inhibition of HIF prolyl-hydroxylase, an α-KG-dependent dioxygenase [79]. This leads to HIF1α activation and consequent up-regulation of HREs target genes involved in angiogenesis, such as VEGF, VEGFR1/2, PLGF-A, PDGF-B, FGF, MMPs, and Angiopoietin-1/2 [80]. The second mechanism involved activation of the Succinate Receptor 1 (SUNCR1), a G-protein coupled receptor for succinate. Upon binding to succinate, SUNCR1 up-regulates VEGF by activating ERK1/2 and STAT3 signaling in a HIF1α-independent manner [13]. Accordingly, succinate may exert a pro- or anti-angiogenic effect and tumor metastasis depending on the cellular context of succinate accumulation.

### Lymphangiogenesis leading to intravasation into lymphatics systems

Emerging evidence indicates that lymphatic vessels density in the vicinity of primary tumors correlates with lymph node metastasis. Mechanistic studies highlighted that HIF-1α is a major transcription factor that induces lymphangiogenesis via mediating VEGF-A/-C expression [81].

#### Bile acids

Lymph nodes present a unique microenvironment enriched with fatty acids. A recent study showed that adaptation of melanoma to lymph node microenvironment requires a shift to fatty acid oxidation. Interestingly, lymph node metastatic melanoma acquired the ability to produce bile acids from cholesterol through CYP7A1 [82]. Bile acids bind to vitamin D receptor and activate yes-associated protein (YAP), which in turn mediated expression of fatty acid oxidase (FAO) and enabled metabolic adaptation in metastatic melanoma extravasated to the lymph node. Besides, YAP forms a complex with HIF-1α in the nucleus and sustains HIF-1α stability to induce its downstream genes including VEGFs and glycolysis under hypoxia [83]. Targeting these metabolic adaptations might help prevent lymphatic metastasis.

### Survival of circulating tumor cells (CTCs) in vasculature transition or fluid circulation

Following invasion into the vasculature, the next step of metastatic cascade is survival in the circulation as circulating tumor cells (CTCs). Few CTCs survive in circulation. CTCs must adapt to environmental stresses, including shear forces, oxidative stress, and escape immunosurveillance to survive [3].

#### Oxidative stress

Detachment of cells from the extracellular matrix is associated with increased oxidative stress. Piskounova et al. [84] systematically evaluated metabolic status in melanoma cells in subcutaneous tumors and in metastatic nodules, showing that the latter had lower GSH/GSSG ratio and increased ROS levels. Administration of antioxidants in vivo promotes tumor metastasis, without any effect on growth of primary tumors, suggesting that oxidative stress represents a major barrier for CTCs. Metastasized melanoma cells have altered expression of folate metabolism enzymes, showing increased NADPH-regenerating enzymes ALDH1L2 and MTHFD1, together with suppression of NADPH-consuming MTHFR. This conserved NADPH, which provided reducing power to regenerate GSH and protected against ROS to promote CTCs survival [84]. Melanoma cells also increased fatty acid oxidation to overcome metabolic stress in CTCs. Li et al. [85] found that ROS induced ERK2-dependent mitochondrial translocation of Nur77, where Nur77 bind and maintained the activity of TPβ, a rate-limiting enzyme of FAO under oxidative stress. This prevented the depletion of NADPH, restricted a surge in ROS, maintained ATP levels and promoted survival of melanoma CTCs. Together, rewiring of metabolic pathways to combat oxidative stress for CTCs survival, and targeting antioxidative mechanisms might be a promising approach to suppress metastasis.

### Extravasation into “fertile soil” at distant premetastatic niches

CTCs in circulation can potentially be deposited at any distant organs; nevertheless, it is observed that CTCs exhibit tropism and tend to form metastasis in specific organs. Both passive extravasation and active homing contributes to tumor extravasation, the first step in metastatic colonization.

#### Nucleotides

Adenine nucleotides play a potential role in extravasation. Tumor cells activated platelets release dense ATP granules, which activates endothelial purinergic P2Y_2_ receptors and induces the opening of the endothelial BM. This is a critical step necessary for transmigration of tumor cells through endothelial cell layer shortly after vascular arrest. Dihydropyrimidine, apart from promoting EMT, is involved in extravasation. Depletion of DYPD reduced ability of HMLER-Twist-ER cells to enter the mouse lung by 95%, a process measured by GFP staining that was significantly rescued by ectopically expression of mouse DPYD (mDPYD) [61].

#### Glycolysis

Tumor cells-released exosomes participate in metastatic cascade, as they encapsulate prometastatic signaling molecules and deliver them to distant organs. Exosomes release is energy intensive and requires aerobic glycolysis. Pyruvate kinase isozyme type M2 (PKM2), which is up-regulated in tumor cells, modulates exosomes secretion by direct phosphorylation of SNAP-23 (Ser95), a key step for synaptosome/ SNARE complex formation and exosome release [86]. Indeed, the deregulation of PKM2 has been shown to promote metastasis in different settings [87].

### Metastatic colonization

The final stage of metastasis involves outgrowth of cancer cells in distant organs. Metastasis initiating-cells (MIC) or cancer stem cells (CSC) are critical in this process, as they possess metabolic versatility to adapt to the microenvironment, and the ability to self-renew and sustain tumorigenesis; non-CSCs could not initiate tumorigenesis. CSCs have demonstrated distinct metabolic programs involved in the maintenance of stemness characteristics. Outgrowth of a metastatic colony also involved reversion of cells to an epithelial phenotype in a process known as the mesenchymal-epithelial transition, and analogous to EMT, involved extensive metabolic rewiring.

#### Fatty acids

Pascual et al. [88] identified a role of fatty acid metabolism in regulation of MICs/CSCs. CD36 is a fatty acid receptor that facilitates import of long-chain fatty acids. A subset of CD44^high^/CD36^high^ cells with metastatic capacity was isolated from oral carcinoma. CD36^high^ CSCs consumed high levels of environmental palmitic acid, and promoted tumor-initiating ability at distant sites. Metastasis of CD36^high^ CSCs was induced by palmitate or high-fat diet, while CD36 blockade with neutralization antibodies impairs tumor metastasis. It is likely that the increased utilization of fatty acids, an energy-dense fuel, could increase the survival of CSCs in distant metastatic niche [89].

#### cNMP

Up-regulation of phosphodiesterase (PDE) in CSCs correlates with increased tumorigenesis, poor prognosis, and tumor metastasis [90]. PDEs are the members of a highly conserved superfamily of enzymes that degrade canonical and noncanonical cyclic nucleotides (cNMPs) [91]. PDEs work in conjunction with adenylate/guanylate cyclases to regulate secondary messengers of G protein-coupled receptor signaling [92]. PDE5 inhibition increased cGMP and cAMP, which stimulates to cAMP/PKA signaling to induce differentiation of CSCs to non-stem tumor cells [90]. Inhibition of PDE5 using a chemical inhibitor has been shown to elevate cGMP which activated cGMP-dependent PKG to attenuate TAZ-mediated gene transcription and suppressed stemness in prostate CSCs [93]. PDE3 also plays a role in CSCs. In pancreatic cancer cells expressing 67-kDa laminin receptor-dependent cGMP inducer, a PDE3 inhibitor in combination with epigallocatechin-3-O-gallate induced cGMP, which impair CSCs properties [94]. In a separate study, cGMP was shown to suppress CD44^high^ pancreatic CSCs [95]. PDEs thus regulates CSCs via modulating cNMPs.

#### Lactate

Lactate dehydrogenase-A (LDHA) is up-regulated in multiple cancers and is important for CSCs [96]. LDHA catalyzes NADH-dependent reduction of pyruvate to lactate and regenerates NAD^+^ required for sustaining glycolysis [97]. Blockade of fermentative glycolysis diminished tumor initiating capacity of CSCs. Consistently, knockdown or pharmacological inhibition of LDHA suppressed cancer stemness, as determined by tumorsphere assay and decreased CD24/44 expression [97].

#### Fructose

Dysregulation of fructose metabolism was found to play a major role in the metastatic colonization of the liver in CRC patients [98]. Aldolase B, which catalyzes reversible conversion of fructose-1,6-bisphosphate to glyceraldehyde-3-phosphate and dihydroxyacetone phosphate, is overexpressed in metastatic lesions as compared to primary tumors [99]. Aldolase B induced incorporation of fructose into glycogen and lipids, both essential for sustaining highly proliferative cells. Aldolase B knockdown in CRC cell lines suppressed liver metastases following cecal transplantation [100]. Moreover, dietary fructose restriction diminished tumor metastasis but had no effect on the primary tumor. Aldolase B is commonly up-regulated in liver metastasis from several cancers [100], and targeting Aldolase B or fructose restriction might be useful for suppressing outgrowth of liver metastasis

## Metastatic signaling-mediated regulation of metabolic gene expression and activity in cancer

### Transcriptional factors

Transcriptional factors modulate expression and activity of metabolic genes directly by regulating their transcription or indirectly by deregulation of metastatic cascades. For example, EMT-associated transcriptional factors (SNAIL, TWIST, and ZEB) and HIFs can mediate rewiring. WNT/SNAIL signaling inhibits mitochondrial respiration by repressing cytochrome C oxidase (COX), the terminal enzyme of mitochondrial respiratory chain, thereby inducing the glycolytic switch [101]. SNAIL also targets fructose-1,6-bisphosphatase (FBP1), a rate-limiting enzyme which catalyzes splitting of F1,6BP into fructose 6-phosphate and inorganic phosphate in gluconeogenesis. The Snail-G9a-Dnmts complex induces promoter methylation and transcription silencing of FBP1, thus promoting glycolysis and inhibiting mitochondrial respiration. A recent study also revealed that ZEB1 could occupy promoter region of FBP1 and attenuate its expression [102]. Yang et al. [103] also showed that TWIST overexpressing cancer cells have up-regulated levels of glycolytic enzymes including LDHA, PKM2, HK2, and G6PD.

HIF signaling drives multiple steps of metastatic cascades [104]. HIF1 reduces mitochondria biomass and upregulates PKM2 to suppress oxidative respiration [105]. HIF1α induces pyruvate dehydrogenase kinase 1 (PDK1), which phosphorylates and inhibits pyruvate dehydrogenase (PDH) to block entry of acetyl-CoA into Krebs cycle, thereby repressing mitochondria respiration [105]. Prolonged hypoxia up-regulates expression of GLUT1, a glucose transporter, via HIF-1 [105]. These results suggest that EMT transcription factors and HIF1 conferred a glycolytic phenotype in cancer cells to trigger metastasis.

### RTK signaling

Receptor tyrosine kinases (RTKs) are overexpressed in cancers. Elevated RTKs are associated with the activation of downstream signaling molecules including MAPK, JAK/STAT, and PI3K/Akt that promoted cancer aggressiveness and metastasis [106]. With respect to the metastatic cascade, AKT, for example, enhances glycolysis flux and activates pentose phosphate pathway to generate NADPH to antagonize increased ROS in metastasis. AKT also promotes inhibition of PDH, thus favouring LDH-mediated biosynthesis of lactate key to maintaining cancer stemness. Activation of ERK1/2 or suppression of AMPK/JNK signaling also mediate a metabolic switch from oxidative respiration to glycolysis in cancer cells in order to fulfill energetic and biosynthetic requirements for tumor metastasis [107].

### Integrins signaling

Integrin proteins including ITGA1, ITGB4, and ITGB5, might be receptors of ECM on cell membranes. ITGB4/FAK/glycogen synthase kinase 3β signaling enhances SOX2, a transcription factor involved in EMT induction, and also induces HIF1α to activate glycolytic gene expression including HK2, GLUT1, and LDHA and modulate glucose metabolism [108]. Alternatively, ITGB4 could bind to PD-L1 to promote SNAI1 and AKT/GSK3β signaling, which in turn activates glycolysis and tumor metastasis [109].

### Therapeutic opportunities targeting the metastasis-metabolism crosstalk in cancer

#### Glycolysis inhibitors

Accumulating evidence indicates that inhibition of glycolysis could be effective for the targeting of metastatic cancers [110]. Glycolysis inhibitors targeting hexokinase (HK) includes 2-deoxyglucose (2-DG), Lonidamine (LND) and 3-bromopyruvate (3-BrPA) (Table 1). A mitochondria-targeted LND demonstrated an antimetastatic effect in vitro and in vivo by inhibition of bioenergetics, induction of ROS and inactivation of AKT/mTOR/p70S6K signaling. Glycolysis blockade with 2- DG or 3-BrPA reversed NAD(P)H:quinone oxidoreductase-1 (NQO1)-induced EMT by inhibiting vimentin, Snail, Slug and Twist, and up-regulating E-cadherin, leading to attenuated NQO1-induced glycolysis and metastasis in breast cancer cells [111]. Lonidamine and 2-DG are currently in Phase I-III clinical trials of multiple metastatic cancers [112–114].

**Table 1:** Alleviation of cancer metastasis using metabolic enzyme inhibitors.

Inhibitor	Target gene	Mode of action	Clinical use/trials	NCT	Reference
Glycolysis inhibitors Lonidamine (LND), Mitochondria-targeted lonidamine (Mito-LND), 2-Deoxyglucose (2-DG)	HK1, HK2	LND/Mito-LND induces ROS and autophagy to block lung cancer migration amd invasion. Mito-LND inhibits p-P70S6K and p-AKT to suppress EMT. 2-DG abrogates NQO1/PKLRdependent glycolysis and tumor metastasis by reversing EMT.	Lonidamine (Phase II/III); 2-deoxyglucose (Phase I/II)	NCT00435448 NCT00096707 NCT00188929	[111, 146, 147]
3-Bromopyruvate (3-BrPA)	GAPDH	3-BrPA inhibits GAPDH, acetyl-CoA production and attenuates NQ01/ PKLR signaling axis-enhanced tumor glycolysis and metastasis via EMT.	N.A.	N.A.	[111]
3PO, PFK-158 (3PO derivative)	PFKFB3	3PO treatment leads to PFKFB3 inactivation and lowered lactate levels. Blockage of glycolysis by targeting PFKFB3 could suppress the migration and invasion of HNSCC cells and reduce growth of both primary and metastatic melanoma tumors.	ACT-PFK-158 (Phase I)	NCT02044861	[116–118]
Glycogenolysis and gluconeogenesis inhibitors					
Dexamethasone	G6PC	Dexamethasone restores gluconeogenesis and inhibits HCC growth and angiogenesis by enhancing G6PC and PEPCK expression.	Phase I/II/III	NCT00695201 NCT00403065 NCT00316927 NCT00176293	[138]
Chlorogenic acid, AD4-015	G6PT	G6PT blockers represses tumor metastasis by suppressing the expression of MMP-2 and MMP-9.	Chlorogenic acid (Phase I/II/III)	NCT03751592NCT02245204NCT02728349NCT03758014	[139, 140]
TCA cycle inhibitors:					
AG-120 (Ivosidenib), AG-881, AGI-5198, AGI-5027	Mutant IDH1	IDH1 inhibitors suppressed 2-HG levels in IDH1-mutant cells. IDH1 inhibtors inhibited SNAIL-dependent EMT and invasive ability of IDH1-mutant cells.	AG-120 (Approved for AML) AG-881 (Phase I)	NCT02073994NCT02989857NCT02481154	[125, 148]
AG-221 (Enasidenib), AG-881, AGI-6780	Mutant IDH2	IDH2 inhibitors suppressed 2-HG production in IDH2-mutant cells to inhibit liver progenitor cell expansion, development of premalignant biliary lesions, and progression to metastatic IHCC.	AG-221 (Approved for AML; Phase I/II)	NCT02273739NCT03515512NCT02481154	[125, 149, 150]
Lipid metabolism inhibitors:					
Omeprazole	FASN	Omeprazole significantly decreased cancer cell invasion and metastasis to the lung and the expression of at least two prometastatic genes, MMP-9 and CXCR4.	N.A.	N.A.	[128, 151]
Cholesterol metabolism inhibitors:			N.A.		
Simvastatin, Pravastatin	HMGCR	Statins inhibit cholesterol biosynthesis and protein prenylyation. Pravastatin treatment greatly reduced the occurrence and extent of spontaneous lung metastasis by decrease the expression of several MMPs including MMP-2, pro-MMP-2, TIMP-2, MMP-14, and MMP-9.	Simvastatin (Phase II/III); Pravastatin (Phase II/III)	NCT03324425 NCT01038154 NCT00433498 NCT01418729	[60, 129, 152]
SAM cycle inhibitors:					
DZNep, Adenosine dialdehyde	SAHH	DZNep and adenosine dialdehyde increased SAH-to-SAM ratio and blocked EZH2-mediated H3K4me3 and transcription of SNAIL and PRC2, leading to reversal of EMT.	N.A.	N.A.	[131, 132]
Nucleotide metabolism inhibitors:					
MY-5445, Sildenafil, Tadalafil	PDE5	Accumulation of cGMP by PDE5 inhibition upregulates cAMP-dependent PKA activity resulting in a reduction of CSCs involved in metastasis and resistance development.	Sildenafil (Phase I/II/III); Tadalafil (Phase II)	NCT02466802 NCT01817751NCT00142506	[90]

*N.A.* not applicable.

Alternative approaches target PFK1 that catalyzes the second rate-limiting step in glycolysis. PFK1 is allosterically activated by fructose-2,6-bisphosphate, a product of 6-phosphofructo-2-kinase/fructose-2,6-biphosphatases (PFKFB1-4). PFKFB3 isoform, which has greater kinase activity than phosphatase activity, is overexpressed in cancer, thereby increasing fructose-2,6-bisphosphate and allosteric activation of PFK1 [115]. 3PO, a PFKFB3 inhibitor, blocked glycolysis and inhibited cell migration/invasion of HNSCC cells [116]. 3PO treatment of endothelial cells also exerts an antimetastatic effect through promoting cell quiescence and tumor vessel normalization [117, 118]. ACT-PFK-158, a derivative of 3PO, is under Phase I clinical trials for advanced solid malignancies (Table 1) [119]. Glycolysis inhibitors thus holds promise in the reversal of metastatic cascades in cancer.

#### IDH1/2 inhibitors

Inhibition of IDH1/2 currently in Phase I/II/III clinical trials has been pursued as a strategy to suppress the production of oncometabolite 2-HG (Table 1) [120–124]. A series of mutant IDH1^R132H^ inhibitors have been reported to inhibit mutant IDH1^R132H^ in a nanomolar range and exhibited selectivity over wildtype IDH1 [9]. Treatment of mutant IDH1^R132C^-expressing hepatoblasts with AGI-5027 (ML309) attenuated 2HG, inhibited liver progenitor cells and restored cell differentiation to halt progression to metastatic intrahepatic cholangiocarcinoma (IHCC) [125]. Besides, treatment of human fibrosarcoma HT1080 cells expressing heterozygous IDH1^R132C^ mutation with AGI-5198 attenuated mTORC1/2 activity, whose oncogenic activation stimulates angiogenesis and metastasis [126]. These proof-of-concept studies indicate that targeting mutant IDH1/IDH2 has potential clinical application as a differentiation therapy to suppress cancer metastasis.

#### Lipid metabolism inhibitors

Endogenous FA biogenesis is tandemly activated by ACC and fatty acid synthase (FASN), which constitutes a metastatic stimulus that drives tumor progression [127]. Omeprazole, a proton pump inhibitor, was shown to inhibit the thioesterase domain of FASN and prevent release of free palmitate from acyl carrier protein. The blockade of FASN by Omeprazole reduced palmitate and dose-dependently inhibited breast cancer cell invasion and metastasis [128].

#### Cholesterol metabolism inhibitors

HMG-CoA reductase catalyzes the conversion of HMG-CoA to mevalonate, the rate limiting step of cholesterol biosynthesis. Statins, inhibitors of HMG-CoA reductase, have been shown to inhibit cell viability, stemness, tumor growth and metastasis through modulation of Sonic Hedgehog (Shh)-related gene expression [129, 130]. Pravastatin treatment greatly reduces the occurrence and extent of spontaneous lung metastasis by repressing MMPs, suggesting a beneficial effect of statins in slowing tumor progression and metastasis [60].

#### SAM cycle inhibitors

SAM blockers inhibit DNMTs and HMTs activities and will likely influence DNA and histone methylation-induced metastasis. SAH hydrolase catalyzes the hydrolysis of SAH into adenosine and homocysteine. As SAH caused by-product inhibition of DNMTs and HMTs, targeting SAH hydrolase compromises DNMTs/HMTs activities. DZNep, a SAH hydrolase inhibitor, blocks EZH2-mediated H3K4me3 and transcription of SNAIL and PRC2, leading to reversal of EMT [131]. Adenosine dialdehyde is another inhibitor of SAH hydrolase, and it down-regulated MMP-9 expression and invasion of cancer cells [132].

#### Nucleotide-metabolic inhibitors

Targeting PDEs can regulate second messengers of G protein-coupled receptor signaling involved in metastatic cascades [92]. MY-5445, a PDE5 inhibitor, induced cGMP and PKA signaling, resulting in a reduction of CSCs involved in metastasis [84]. Another PDE5 inhibitor, Sildenafil, approved by FDA for pulmonary arterial hypertension, has been tested in Phase I/II/III trials for advanced solid tumors. Sildenafil was shown to suppress postoperative metastasis by targeting surgery-induced myeloid derived suppressor cell (MDSC)-dependent inhibition of NK cell cytotoxicity (Table 1) [133–136]. Sildenafil down-regulated ARG1, IL4Ra and ROS to inhibit MDSC-mediated suppression of NK and T cells. Targeting PDE5 with Tadalafil also alleviated MDSC and NK cells-associated metastasis in phase II trials of metastatic head and neck squamous cell carcinoma and oesophagus cancer (Table 1) [136, 137]. Thus, PDE5 is a potential target for antimetastatic therapy.

#### Glycogenolysis and gluconeogenesis inhibitors

Inhibition of glucose-6-phosphate translocase (G6PT), which transports G-6-P from cytoplasm into ER lumen, has been pursued as a strategy to regulate glycogenolysis and gluconeogenesis [138]. Inhibition of G6PT antagonizes tumor metastasis through the inhibition of MMP2 [139], MMP9 [140], and angiogenesis. G6PT inhibitors including chlorogenic acid, mumbaistatin, and AD4-015 are currently under evaluation in cancer treatment. Chlorogenic acid is being tested in Phase I/II/III trials for advanced lung cancer and glioblastoma (Table 1) [141–144].

## Conclusion and 5-year view

**Fig. 2:**
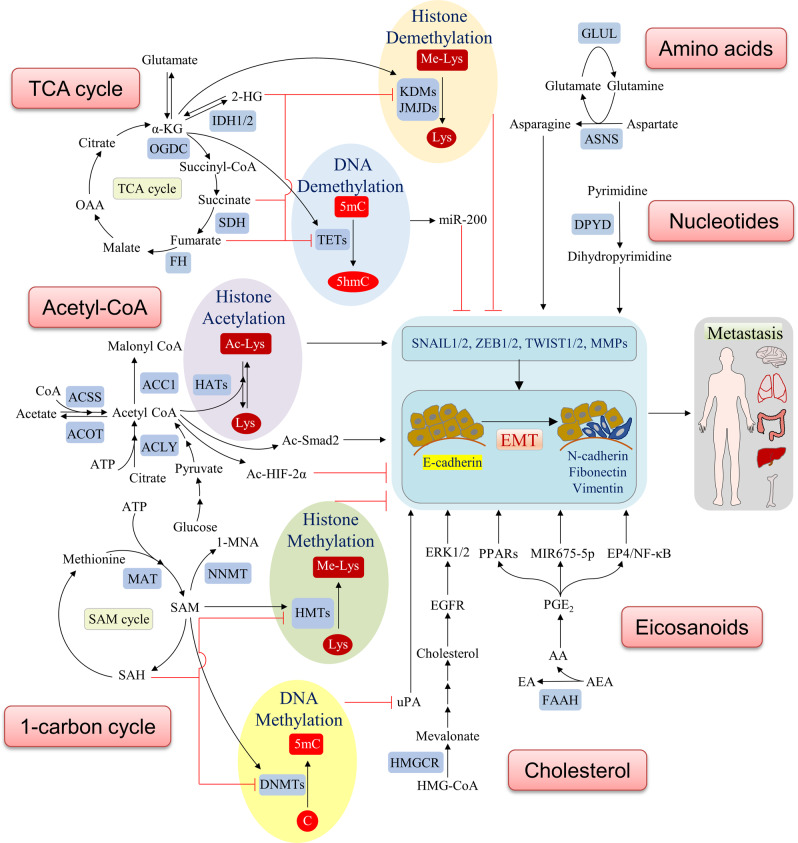
Molecular mechanisms of metabolites-mediated regulation of EMT. Many metabolites contribute to rewiring of metastatic pathway in cancer progression.

Crosstalk between cellular metabolism and metastatic cascades are the consequences of adaptation to unique nutrition requirement for invasion/metastasis and to overcome environment stress in the circulation and metastatic niche. As we have summarized, the tumor metastatic proteome and transcriptome are dynamically modulated by the metabolome. Metabolome-induced signaling cascades may drive tumor aggression and metastasis via diverse pathways involved in every step of the metastasis cascade (Figs. 1 and 2). Here we have outlined potential strategies targeting the crosstalk between metastatic signaling and cell metabolism for selective blockage of metastasis (Tables 1 and 2). Currently, much experimental research involved in vitro cell culture systems, which poorly reflects the complexity of metastasis in vivo that involved not only metastatic tumor cells, but also stromal and immune components. In the future, preclinical studies need to take into consideration the tumor microenvironment to validate potential targets, which ultimately will contribute to identification of novel molecular targets for suppression of metastasis.

**Table 2:** Regulation of cancer metabolism by antimetastatic drugs.

Inhibitor	Target gene	Mode of action	Clinical use/trials	NCT	Reference
Transcription factor inhibitors:					
Tigecycline	MYC	Tigecycline inhibits cell migration/invasion. It also suppresses mitochondrial OxPhos, glycolysis and extracellular acidification in various neoplasms.	Phase I	NCT01332786	[153, 154]
TH-302 (Evofosfamide), EO9 (Apaziquone)	HIF-1α	HIF-1α inhibitors blocked a shift of OxPhos to anaerobic glycolysis for decreasing ROS levels, and eventually inhibited metastatic colonization to the lungs.	TH-302 (Phase I/II/III); EO9 (Phase I/II/III)	NCT02076230 NCT02093962 NCT01746979 NCT01373398 NCT00141531 NCT03224182	[155–157]
Fatostatin	SREBP1/2	Fatostatin lowers expression of SREBP-regulated enzymes for fatty acid and cholesterol synthesis of FA, and inhibited prostate tumor growth and distant lymph node metastasis.	N.A.	N.A.	[158]
Kinase inhibitors:					
Cetuximab, Erlotinib Panitumumab,	EGF-EGFR	Anti-EGFR antibodies and inhibitors are commonly used in metastatic cancers and they reversed the Warberg effect by inhibiting HIF-1-regulated glycolysis.	Approved for multiple cancers	N.A.	[106]
Bevacizumab (Avastin), Sunitinib (Sutent)	VEGF-VEGFR	Anti-VEGF antibodies are commonly used in metastatic cancers and they also suppressed glucose metabolism.	Approved for multiple cancers	N.A.	[106, 159–161]
Trametinib	MEK	Trametinib blocks RAS-RAF-MEK-ERK MAPK cascades mediated-EMT and suppresses pHi and glycolysis in response to hypoxia.	Approved for Braf mutant melamona; (Phase I/II)	NCT03299088 NCT03825289 NCT02070549 NCT03428126	[162, 163]
Dasatinib,Saracatenib	SRC	Saracatinib and dasatinib suppress migration of mesenchymal-like HNSCC cells. SRC inhibitor blocks c-Myc translation and glycolysis.	Approved for CML and resistant ALL; (Phase I/II/III)	NCT00882583 NCT02116712 NCT00669019 NCT00607594	[159, 164–166]
Integrin inhibitors:					
Cilengitide	αvβ3/αvβ5- integrin	Cilengitide treatment of breast cancer bone metastasis resulted in a significant reduction in fluorine-18 fluorodeoxyglucose uptake.	Cilengitide (Phase II)	NCT01517776 NCT00082875 NCT00842712 NCT00103337	[167, 168]
Epigenetic regulators					
Sodium butyrate, SAHA (Vorinostat), LBH589 (Panobinostat), JNJ-26481585 (Quisinostat)	HDACs	HDAC inhibitors has been shown to reduced glucose uptake, glycolytic flux and lactate metabolism. Inhibition of HDAC11, a binding/transport protein for free fatty acids/acyl-CoA also impact fatty acid metabolism.	Vorinostat (Approved for Cutaneous T-cell lymphoma), Panobinostat (Approved for Multiple myeloma)	NCT00002796 NCT00005639 NCT00387530	[169–171]

*N.A.* not applicable.

With numerous drugs targeting metabolism in clinical development, we will have the tools to be able to effectively target these abnormalities in cancer in the next 5 years. L-asparaginase, novel drugs inhibiting asparagine, for instance, is approved by FDA for ALL and undergoing phase II trials for metastatic pancreatic cancer [145]. A caveat of targeted therapies, as exemplified by the application of receptor tyrosine kinase inhibitors, is that they are helpful only when their target(s) are the main drivers of metastatic carcinogenesis. To fully realize the potential of metabolic/antimetastatic modulators, future clinical trials should incorporate analysis of biomarkers to unravel metastatic proteome, transcriptome and metabolomic markers that allow the selection of subsets of patients that might benefit most from targeted treatments. Given the extensive association between metastatic cascade and metabolic pathway, perhaps the combination approaches involving metastatic signaling and metabolic regulators may achieve synergistic metastasis inhibition. Development of rationale drug combinations involving metabolic inhibitors, antimetastatic agents, together with traditional chemotherapeutics will likely have the greatest impact on future cancer management.
